# Isolation and Characterization of Chitosan-Producing Bacteria from Beaches of Chennai, India

**DOI:** 10.1155/2012/421683

**Published:** 2012-08-05

**Authors:** Kuldeep Kaur, Vikrant Dattajirao, Vikas Shrivastava, Uma Bhardwaj

**Affiliations:** ^1^Department of Biotechnology, School of Basic Sciences, Arni University, Indora, H.P., Kathgarh 176401, India; ^2^CRM Department, Serum Institute of India Limited, Hadapsar, Pune 411028, India

## Abstract

Chitosan is a deacetylated product of chitin produced by chitin deacetylase, an enzyme that hydrolyses acetamido groups of N-acetylglucosamine in chitin. Chitosan is a natural polymer that has great potential in biotechnology and in the biomedical and pharmaceutical industries. Commercially, it is produced from chitin via a harsh thermochemical process that shares most of the disadvantages of a multistep chemical procedure. It is environmentally unsafe and not easily controlled, leading to a broad and heterogeneous range of products. An alternative or complementary procedure exploiting the enzymatic deacetylation of chitin could potentially be employed, especially when a controlled and well-defined process is required. In this study, 20 strains of bacteria were isolated from soil samples collected from different beaches of Chennai, India. Of these 20 bacterial strains, only 2 strains (S3, S14) are potent degrader of chitin and they are also a good producer of the enzyme chitin deacetylase so as to release chitosan.

## 1. Introduction

Chitin, a homopolymer of *β* (1,4)-linked N-acetyl-glucosamine, is one of the most abundant, easily obtained, and renewable natural biopolymers, second only to cellulose [1]. Chitin is considered the second most plentiful organic resource on the earth next to cellulose and is present in marine invertebrates, insects, fungi, and yeasts. Chitin and its derivatives have high economic value owing to their versatile biological activities and agrochemical applications [2, 3]. Chitin is not soluble in water or in the majority of organic solvents. However, chitosan, prepared from chitin (usually of crab or shrimp shell origin) through chemical N-deacetylation, is water soluble and possesses biological properties such as high biocompatibility and antimicrobial activities. Chitosan is widely used in medical applications including antitumor therapy and cholesterol control, in medicinal membranes, wound dressings, and controlled-released medicinal materials [4]. Recently, chitosan has also been used as a natural substance for the enhancement of seed germination and plant growth and also as an ecologically friendly biopesticide to boost the innate plant defense mechanisms against fungal infections. At present, chitosan is produced by the thermochemical deacetylation of chitin. Thus an envirofriendly bacterial strains-mediated method can be successfully used for the enzymatic deacetylation of chitin, especially when a controlled and well-defined process is required.

**Figure 1:**
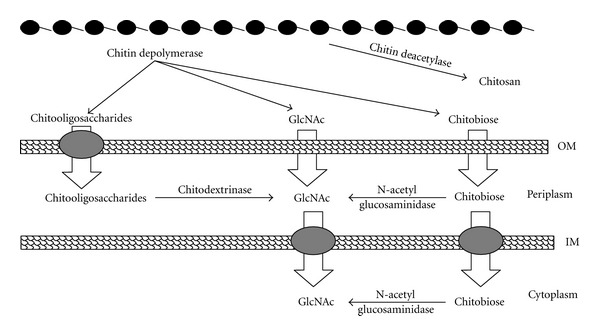


Chitin deacetylase (CDA), first identified and partially purified from extracts of the fungus *Mucor rouxii* [5], is the enzyme that catalyzes the conversion of chitin to chitosan by the deacetylation of N-acetyl-D-glucosamine residues (Figure 1). The presence of this enzyme activity had also been reported in several other fungi [6] and in some insects [7]. The enzyme is an acidic glycoprotein of ~75 kDa with 30% (w/w) carbohydrates, exhibits a remarkable thermal stability at their optimum temperature (50°C), and displays a wide range of pH optima [8]. One of the interesting properties with biotechnological application is that they are not inhibited by acetate, a product of the deacetylation reaction. 

Presently, chitosan is produced from chitin by a chemical NaOH pyrolysis method. This method has some problems, such as environmental pollution, high energy consumption, and poor quality of the resulting chitosan. Use of CDA-producing fungi for chitin N-deacetylation theoretically could circumvent these problems, but the CDA producing capabilities of most fungal strains are low and their fermentation requirements are complicated. A search has therefore been initiated to find a more suitable strain of CDA-producing bacteria to replace the current fungal strains [9]. Biotransformation of chitin to chitosan by bacteria can be used in an economical and environmentally friendly process. Bacteria are easier and faster than fungi to grow in a large-scale fermentation system. Additionally, bacteria can be utilized without the necessity of purifying the enzyme. This paper reports the isolation and identification of bacterial strains from soil of beaches of Chennai that produces CDA which transform chitin to chitosan.

## 2. Material and Methods

### 2.1. Sources of Media and Analytical Chemicals

All chemicals used were of analytical grade. Media and chemicals used in this study were purchased from HiMedia, Qualigen, and SD Fine Chemicals, India.

### 2.2. Soil Sampling and Analysis

Soil samples were collected from different beaches of Chennai, India using a sterile scalpel. Samples were stored individually in sterile polythene bags. Samples were analyzed for organic carbon, available phosphorus, and for microbial population. The organic C in the soil samples was 1.25%. Percentage availability of total phosphorus was 60. The pH of the soil was in the range 6.00–6.50.

### 2.3. Isolation of Bacterial Isolates

Cultivable bacterial strains were isolated using initial screening in normal saline (0.9%). Population counts of soil samples were determined by dilution plating on NA plate with vortexing at every dilution step. Plates were incubated in B.O.D incubator with 80% relative humidity at 30 ± 0.2°C for 24 hrs. Colonies were counted and restreaked on NA. Pure cultures were preserved as glycerol stock and stored at −70°C.

### 2.4. Characterization of Isolates

#### 2.4.1. Morphological Characterization

Morphological characteristics, namely, colony morphology (color, shape, margin, elevation, and surface) cell morphology (shape, gram reaction, and arrangement) of recovered isolates were studied.

#### 2.4.2. Biochemical Characterization

The various bio-chemical tests, namely, IMViC, triple sugar iron agar, nitrate reduction test; urease test and catalase test were carried out according to [10], for characterization of isolates.

### 2.5. Screening for Chitinase Degrading Activity

The single bacterial colonies were screened on selective medium (chitin 1%, NaNO_3_ 0.2%, K_2_HPO_4_ 0.1%, KH_2_PO_4_ 0.1%, MgSO_4_ 0.05%, P and N 0.05%, and agar 2%) and cultured for 2 more days at 30°C. Bacteria with chitin degrading activity were further screened for CDA. 

### 2.6. Screening of Cultures for CDA

Solution of p-nitroacetanilide was prepared by dissolving 5 g of p-nitroacetanilide in 100 mL of ethanol. Strips of Whatman #1 filter paper were cut to size of 5 cm × 1.0 cm. These strips were immersed in the solution of p-nitroacetanilide, removed, and air-dried. This was repeated thrice to impregnate the strips with a sufficient concentration of p-nitroacetanilide. The dried strips were used for the test. Test tubes containing 5 mL of presterilized medium of composition: 1 g of yeast extract, 0.4 g of ammonium sulfate, and 0.15 g of potassium dihydrogen phosphate (pH 8.0) were inoculated with organisms from individual colonies of the isolates and kept one test tube as control. Test tubes were incubated at 25°C for two days. After incubation, 2 mL aliquots were transferred to another set of sterile test tubes containing the diagnostic strips. These tubes were then incubated at 25°C for 12–24 hours. After incubation, the development of yellow color in the strip indicates the presence of deacetylase in respective bacterial isolate [11–13].

### 2.7. Transformation of Chitin to Chitosan by Isolates (S3, S14)

The production media for CDA (1 g of yeast extract, 0.4 g of ammonium sulfate, and 0.15 g of potassium dihydrogen phosphate (pH 8.0) containing 50 mg of chitin) was used as fermentation medium. 250 mL capacity flasks with 50 mL of fermentation medium were taken. The flasks were inoculated with 1 mL of 0.1 O.D._600_ suspensions of the positive isolates. The one flask was not inoculated and used as control. All flasks were incubated on rotary shaker at 25°C for two days. After incubation, each flask was taken for chitosan recovery.

### 2.8. Recovery of Chitosan from Production Media

The fermented broth from each flask was centrifuged at 12000 rpm for 15 minutes. The supernatant was discarded. The pellet contained mixture of bacteria, chitin, and chitosan. To each of these pellets was added 10 mL of 0.1 N NaOH. The contents were mixed thoroughly and taken in separate clean test tubes that were autoclaved for 15 minutes. The tubes were then allowed to come to room temperature. Most of the cells were solubilized during the alkaline treatment. The tubes were again centrifuged at 12000 rpm for 15 minutes. The supernatants were carefully removed and pellets containing chitin, chitosan, and small amount of cell debris were mixed with 10 mL of 2% acetic acid and mixtures were taken in clean test tubes that were left on a shaker overnight at room temperature to solubilize chitosan in 2% acetic acid. The contents of the above tubes were again centrifuged at 12000 rpm for 15 minutes. Pellet was discarded and 10 mL supernatant was collected and the presence of chitosan in it was checked by the formation of white precipitate upon neutralization with 1 N NaOH [11].

### 2.9. Qualitative Estimation of Chitosan 

The white precipitate obtained after recovery was centrifuged at 5000 rpm for 15 minutes. It was washed twice with distilled water (pH 7). Then precipitate was resuspended in 0.5 mL of distilled water (pH 7) and this suspension was taken in watch glass. It was allowed to dry at 55°C for 2–4 hours. The dried precipitate was used for the confirmatory test. On the dried precipitate 2-3 drops of iodine/potassium iodide solution were added and mixed and the mixture was acidified with 2-3 drops of 1% H_2_SO_4_. After addition of iodine/potassium iodide solution, the precipitate change color to dark brown and the solution becomes colorless and on addition of sulfuric acid the dark brown color turns to dark purple. This indicates the presence of chitosan [14–16].

### 2.10. Quantitative Estimation of Chitosan

 Once again the precipitate of chitosan was obtained. It was washed twice with distilled water (pH 7) and was resuspended in 1 mL of distilled water (pH 7). The weights of two dried, clean petriplates were taken. In that petriplates, 1 mL of chitosan suspension obtained from the isolates was poured. Petriplates were kept at 55°C for 2–6 hours for drying. After drying, plates were again weighed.

## 3. Results

### 3.1. Isolation and Characterization of Isolates

In total, 20 bacteria were isolated from fresh soil samples. They exhibited wide morphological variation (Table 1). Bacterial morphotypes were selected on the basis of their color, morphological characteristics, namely, colony morphology (shape, margin, elevation, and surface) and cell morphology (gram reaction, cell shape, and arrangement) according to Bergey's Manual of Systematic Bacteriology [17].

### 3.2. Screening of Chitin Degrader

**Figure 2:**
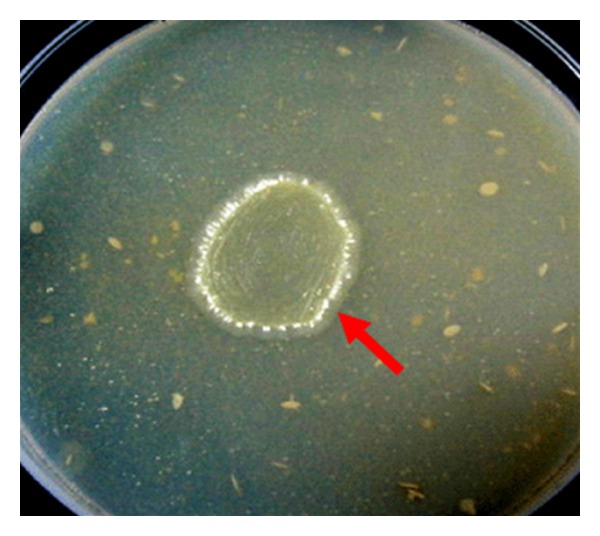
Chitinolytic activity was studied using chitin agar.

Out of 20 pure bacterial isolate, only two bacterial cultures, one of them was Gram-positive rods (S3) and other was Gram-negative rods (S14) are chitin degraders strains (Figure 2) determined by growth on the selective medium. S3 is Gram-positive, endospore-forming, rod-shaped bacterium with catalase-positive, nitrate reduction-positive, indole-positive, capable of starch and gelatin hydrolysis, MR-negative, and VP-negative whereas S14 is a Gram-negative rods with catalase-positive, VP-positive, indole-negative and MR-negative.

### 3.3. Screening of Cultures for CDA

 Above 2 isolates are potent chitin degraders, so it was presumed that they would also produce the enzyme chitin deacetylase so as to release chitosan, conforming to earlier reports of Alexander in 1985 [18]. Therefore, these isolates were screened for their chitin deacetylase activity using the diagnostic strip test (Table 2) for conversion of p-nitroacetanilide by the enzyme, which is itself believed to be novel.

### 3.4. Production of Chitosan

the results of this study in which the two bacterial isolate S3 and S14 were cultivated in a production medium containing chitin are presented in Table 3. The fermented broth after specified incubation period was tested for the presence of chitosan and results in Table 3 indicate that S3 and S14 release chitosan from raw chitin. The precipitate obtained was indeed chitosan that was confirmed by its reaction that gave rise to a dark purple coloration.

### 3.5. Yield of Chitosan

Once it was proved that the isolates release chitosan, it became imperative that the yield was also determined. This was done by a gravimetric method. The results of which are presented in Table 4.

## 4. Discussion

Chitosan has a great potential in biotechnology especially in the biomedical and pharmaceutical industries [19]. Chitosan is widely used in medical applications including antitumor therapy and cholesterol control, in medicinal membranes, wound dressings, and controlled-released medicinal materials [4].

Chitosan is produced from chitin via a harsh thermochemical procedure. This process shares most of the disadvantages of a severe chemical procedure; it is environmentally unsafe and not easily controlled, leading to a broad and heterogeneous range of products. Also, the chitosan manufactured by chemical methods gives the product of inferior quality with respect to its properties like viscosity, molecular weight, and degree of deacetylation. An alternative procedure that would exploit the enzymatic deacetylation of chitin was using chitin deacetylase. So the use of chitin deacetylase for the preparation of chitosan polymers and oligomers offers the possibility of the development of an enzymatic process that could potentially overcome most of these drawbacks. The chemical method also produces alkaline wastes that could be minimized with biological degradation of sugar chain [20]. Chitin deacetylase (CDA; EC 3.5.1.41) catalyses the hydrolysis of N-acetamido bonds in chitin to produce chitosan thus generating glucosamine units and acetic acid. The presence of this enzyme activity has been reported in several fungi [21, 22] and insect species [7]. Use of CDA-producing fungi for chitin N-deacetylation theoretically could evade these problems, but the CDA producing capabilities of most fungal strains are low and their fermentation requirements are complicated. So, there is a need to search for a more suitable strain of CDA-producing bacteria to replace the current fungal strains [9]. CDA from other microorganisms mainly bacteria was rarely reported while the numbers of marine bacteria widely distributed in oceanic and estuarine waters are mainly responsible for recycling of nitrogen present in chitinous debris [23]. Earlier it was shown that chitin hydrolysis was carried out by at least two enzymes, a chitinase that mainly produced N, N′-diacetylchitobiose (GlcNAc)2 and a beta-N-acetylglucosaminidase that gave the final product, GlcNAc [20]. Recently Jung et al. [24] described the involvement of CDA genes in the chitin catabolic cascade of Vibrios.

In this study, we have isolated the CDA-producing bacteria from flora of seashore. In total, 20 bacteria were isolated, out of which 2 isolates (S3, S14) showing chitosan degrading ability were screened for their chitin deacetylase activity. Identification of these isolates was also carried out using various physiological and biochemical tests as outlined in the Bergey's Manual of Systematic Bacteriology [25]. Based on the morphological and physiochemical analysis, the isolates S3 and S14 were identified as *Bacillus* sp. and *Serratia* sp., respectively. Some *Bacillus* species with CDA were reported, previously namely; *Bacillus thermoleovorans* [26], acidophilic *Bacillus* sp. [27, 28], and *B. stearothermophilus* [29]. *Serratia* species tested for chitosan susceptibility while there is no report of chitosan production by *Serratia*, only genome sequencing of *Serratia proteamaculans* strain 568 reported the chitin deacetylase gene (EMBL ABV40022.1) [30]. 

 The yield of chitosan by S3 and S14 was 0.16 g/L and 0.1 g/L, respectively, using chitin as sole carbon source. Different amounts of chitosan production from fungi have been reported. We obtained the higher amount of chitosan than Crestini et al. [31] where the yield of isolated chitosan was 0.12 g/L of fermentation medium under liquid fermentation conditions and Ke-Jin Hu et al. reported a 78.3 mg/L yield using PGY salt broth for *A. niger* [32].

Muzzarelli et al. [19] obtained about 1.8 g/L of chitosan with *Absidia coerulea* using a PGY medium, while Davoust and Persson [33] reported a 2.8 g/L yield using glucose, yeast, and mineral media. The yield of chitosan produced in this work was not very high but can be improved with optimization of fermentation conditions that can increase the CDA production to much higher level. So these bacteria can be exploited for biotransformation of chitin to chitosan at industrial scale and proved to be a promising candidate for an economical and environmentally friendly process.

## Figures and Tables

**Table 1:** Colony morphology of recovered isolates of soil samples.

	Isolate	Form	Size	Color	Margin	Elevation	Surface	Opacity	Organism
1	S1	Circular	Small	Dirty white	Entire	Raised	Smooth	Opaque	*Staphylococcus *sp.
2	S2	Circular	Small	Yellow	Entire	Flat	Smooth	Opaque	*Micrococcus *sp.
3	S3	Circular	Small	White	Entire	Raised	Dry	Opaque	*Bacillus *sp.
4	S4	Circular	Small	Dirty white	Entire	Flat	Smooth	Opaque	*Streptococcus *sp.
5	S5	Irregular	Small	Greyish	Senate	Raised	Smooth	Opaque	*Proteus *sp.
6	S6	Circular	Small	White	Entire	Raised	Dry	Opaque	*Bacillus *sp.
7	S7	Irregular	Small	White	Entire	Raised	Smooth	Opaque	*Alcaligens *sp*. *
8	S8	Circular	Small	White	Entire	Raised	Smooth	Translucent	Unidentified
9	S9	Circular	Small	White	Entire	Raised	Smooth	Opaque	Unidentified
10	S10	Circular	Small	Yellow	Entire	Raised	Smooth	Opaque	*Staphylococcus* sp*. *
11	S11	Circular	Small	Dirty white	Entire	Flat	Smooth	Opaque	*Streptococcus *sp.
12	S12	Circular	Small	Yellow	Entire	Raised	Smooth	Opaque	*Micrococcus *sp.
13	S13	Circular	Small	Light green	Entire	Raised	Wrinkled	Opaque	*Pseudomonas *sp.
14	S14	Circular	Small	Red	Entire	Raised	Smooth	Opaque	*Serritia *sp.
15	S15	Circular	Small	Light green	Entire	Raised	Wrinkled	Opaque	*Pseudomonas *sp.
16	S16	Circular	Small	White	Entire	Raised	Dry	Opaque	*Bacillus *sp.
17	S17	Circular	Small	Dirty white	Entire	Raised	Smooth	Opaque	Unidentified
18	S18	Circular	Small	White	Entire	Flat	Smooth	Translucent	Unidentified
19	S19	Circular	Small	White	Entire	Raised	Dry	Opaque	*Bacillus *sp.
20	S20	Circular	Small	Light green	Entire	Raised	Wrinkled	Opaque	*Pseudomonas *sp.

**Table 2:** Results of diagnostic strip test.

Tube type	Organisms inoculated	Initial color of diagnostic strip	Color of the strip after incubation at 25^°^C for 24 hours	Chitin deacetylase activity
B	S3	Colorless	Yellow	**+**
C	S14	Colorless	Yellow	**+**
Control	Not inoculated	Colorless	Colorless	**−**

+: Chitin deacetylase activity present.

**−**: Chitin deacetylase activity absent.

**Table 3:** results of transformation of chitin to chitosan by the isolates.

Flask type	Fermented broth of isolate S3	Fermented broth of isolate S14	Control flask (not inoculated)
Chitosan recovered in	10 ml of 2% acetic acid	10 ml of 2% acetic acid	10 ml of 2% acetic acid
Neutralization of 2% acetic acid with 1 N NaOH	White precipitate observed	White precipitate observed	No precipitate observed
Iodine reaction	Dark purple coloration	Dark purple coloration	**—**

**Table 4:** Results of yield of chitosan by the isolates.

Isolates	Amount of chitosan produced in g/L	% age
S3	0.16	16
S14	0.1	10
